# A ubiquitinome and proteome dataset of the red alga *Gracilariopsis lemaneiformis* under cold stress

**DOI:** 10.1038/s41597-026-07789-w

**Published:** 2026-10-19

**Authors:** Feng Qin, Xiuyu Zhang, Shuangshuang Peng, Jianxin Sun, Di Xu, Weizhou Chen, Xiaonan Zang

**Affiliations:** 1 Key Laboratory of Marine Genetics and Breeding (Ocean University of China), Ministry of Education, Qingdao, 266003 Shandong China; 2 Marine Science Institute, Shantou University, Shantou, 515063 China; 3 Guangdong Provincial Key Laboratory of Marine Biotechnology, Shantou University, Shantou, 515063 China

## Abstract

Low temperature is a major environmental factor constraining the growth and aquaculture performance of the red alga *Gracilariopsis lemaneiformis*, yet systematic protein-level data describing cold stress responses remain limited. Ubiquitination is a key post-translational modification involved in regulating protein stability and turnover during stress adaptation. Here, we present a paired dataset of the global proteome and the ubiquitin-modified proteome of *G. lemaneiformis* under cold stress, generated using a matched experimental design across identical conditions. This paired dataset provides a valuable resource for future investigations of the relationship between protein abundance changes and ubiquitination regulation during cold-stress responses. We further provide detailed technical validation demonstrating high data quality and strong reproducibility among biological replicates. These datasets constitute a valuable resource for investigating ubiquitination-associated protein homeostasis during cold adaptation, facilitating the identification of ubiquitination-regulated substrate candidates under cold stress, and supporting downstream functional studies as well as the breeding of cold-tolerant *G. lemaneiformis* strains.

## Background & Summary

*Gracilariopsis lemaneiformis* is a high-value red alga widely cultivated for agar production. It is also commonly used as a natural feed for abalone and other herbivorous mollusks, and plays important roles in integrated multi-trophic aquaculture and coastal ecological restoration^1–3^. However, its growth and aquaculture performance are highly sensitive to temperature. Although this alga grows optimally at 11–23 °C, exposure to temperatures below 10 °C during winter or cold snaps leads to marked growth inhibition, disruptions in photosynthetic performance and cellular damage. These cold-induced physiological constraints limit biomass accumulation and restrict stable year-round cultivation. Understanding how *G. lemaneiformis* responds to cold stress is critical for deciphering its acclimation mechanisms and facilitating the development of cold-tolerant cultivars.

Post-translational modifications (PTMs) regulate cellular responses to environmental stress by modulating protein stability, activity, localization and interaction networks^4^. Ubiquitination is a crucial PTM in which ubiquitin-activating (E1), ubiquitin-conjugating (E2), and ubiquitin ligases (E3) sequentially collaborate to covalently attach a ubiquitin molecule (composed of 76 amino acids) to the lysine residues of target proteins. Ubiquitination plays a central role in targeted protein degradation and protein homeostasis^5,6^. While ubiquitination has been extensively studied in higher plants^7–9^, research in algae remains comparatively limited. In particular, the molecular mechanisms by which ubiquitin-mediated protein modification contributes to environmental adaptation in macroalgae remain largely unexplored. Research has demonstrated that the ubiquitin-proteasome system may be involved in algal stress responses. For example, ubiquitome profiling of the kelp *Saccharina japonica* under heat stress identified extensive remodeling of lysine ubiquitination on proteins involved in photosynthesis, metabolism and stress defense, highlighting diverse roles of ubiquitination in thermal acclimation of brown macroalgae^10^. A RING-type E3 ubiquitin ligase, PhCUL4, was functionally characterized in *Pyropia haitanensis* and shown to enhance thermotolerance, providing direct evidence that specific UPS components contribute to heat-stress adaptation in marine macroalgae^11^. Similarly, genes associated with the ubiquitin–proteasome system were significantly induced during pathogen infection in the red alga *Pyropia yezoensis*^12^. Furthermore, ubiquitination-related pathways were activated under dehydration stress in the red seaweed *Gloiopeltis furcata*, suggesting an important role of ubiquitin-mediated protein modification in intertidal stress adaptation^13^. These findings suggest that ubiquitination may represent a conserved regulatory mechanism mediating algal adaptation to diverse environmental stresses. However, ubiquitination-associated responses to low temperature in *G. lemaneiformis* remain largely unexplored.

Although ubiquitome profiling has been reported in the brown macroalga *S. japonica* under heat stress^10^, that study mainly focused on global ubiquitination changes in response to high temperature and did not include a matched global proteome dataset for parallel comparison. In contrast, the present study provides paired global proteome and ubiquitin-modified proteome datasets for the red alga *G. lemaneiformis* under low-temperature stress, thereby offering a complementary resource for studying ubiquitination-associated cold-stress responses in red macroalgae. To provide a comprehensive resource for understanding ubiquitin-mediated regulation in *G. lemaneiformis* during cold stress, we generated paired datasets of the global proteome and ubiquitin-modified proteome using LC–MS/MS under a matched experimental design. These datasets capture changes in protein abundance as well as site-specific lysine ubiquitination events under low-temperature treatment, providing a basis for future investigations of abundance- and ubiquitination-level regulation. These resources may support downstream functional studies and the prioritization of candidate ubiquitination-regulated substrates associated with cold-stress responses.

## Methods

### Sample collection and low-temperature treatment

**Fig. 1:**
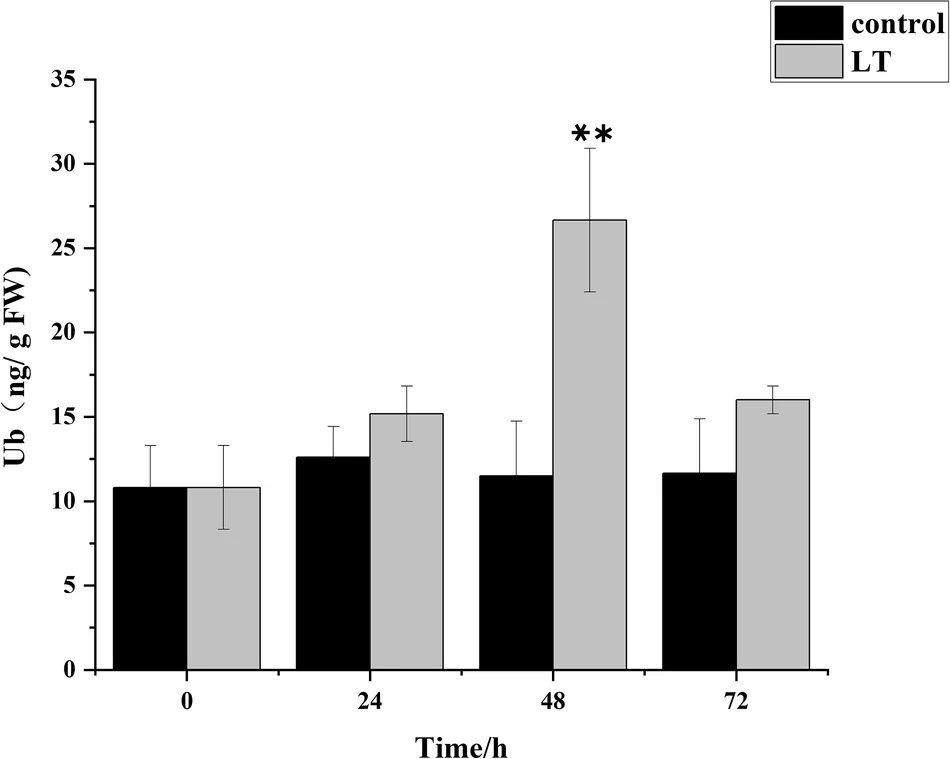
Changes in ubiquitin (Ub) content in *G. lemaneiformis* during low-temperature (LT) treatment. Samples were exposed to 5 °C for 0, 24, 48, and 72 h. Asterisks indicate significant differences compared with the control group (P < 0.01).

Wild tetrasporophytes of *G. lemaneiformis* were collected from the intertidal zone of Fushan Bay, Qingdao, China. Tetrasporophytic thalli were identified and selected under a stereomicroscope based on the presence of typical tetrasporangial structures. Fresh thalli were rinsed thoroughly with sterile seawater to remove surface epiphytes and debris. Algae were maintained in sterile seawater supplemented with f/2 medium at 20 °C under a 12 h light/12 h dark photoperiod with a light intensity of 20 μmol photons m^−2^ s^−1^. After acclimation, healthy branches were randomly assigned to two temperature treatments: (i) 20 °C as the control group (CK), and (ii) 5 °C as the low-temperature group (LT), while all other culture conditions were maintained identically between treatments. The 48-h treatment duration was selected based on a preliminary time-course assay, in which *G. lemaneiformis* samples were exposed to 5 °C for 0, 24, 48, and 72 h (Fig. 1). Ub content showed the most pronounced and statistically significant change at 48 h; therefore, 5 °C for 48 h was selected as a representative time point for proteome and ubiquitinome profiling.

After 48 h of cultivation, algal tissues were collected, quickly frozen in liquid nitrogen, and stored at −80 °C for subsequent protein extraction. Each group contained three biological replicates (n = 3). For each group, the collected tetrasporophytic thalli were randomly divided into three portions of comparable biomass, with 300 mg of tissue used for each replicate. Each replicate consisted of pooled algal material from multiple thalli and was independently processed for low-temperature treatment, protein extraction, and subsequent proteome or ubiquitinome analysis.

### Protein extraction and trypsin digestion

After removal from −80 °C storage, *G. lemaneiformis* samples were thoroughly ground into a fine powder in liquid nitrogen. For each group, four volumes of phenol extraction buffer (containing 10 mM DTT, 1% protease inhibitor, and 50 μM PR619) were added, followed by ultrasonic lysis. An equal volume of Tris-buffered phenol was then added, and the mixture was centrifuged at 5,500 × g for 10 min at 4 °C. The supernatant was collected and precipitated overnight with five volumes of 0.1 M ammonium acetate in methanol. The resulting pellet was washed sequentially with methanol and acetone, then dissolved in 8 M urea. Protein concentrations were quantified using a BCA assay kit, and protein quality was assessed by SDS-PAGE followed by Coomassie Brilliant Blue R-250 staining.

Equal amounts of protein from each sample were subjected to enzymatic digestion, and the volumes were adjusted to be consistent using lysis buffer. Trichloroacetic acid (TCA) was slowly added to a final concentration of 20%, followed by vortex mixing and precipitation at 4 °C for 2 h. The samples were centrifuged at 4,500 × g for 5 min, and the supernatant was discarded. The precipitated proteins were washed with prechilled acetone 2–3 times. After air-drying the pellet, TEAB was added to a final concentration of 200 mM, and the pellet was resuspended by ultrasonication. Trypsin was added at an enzyme-to-protein ratio of 1:50 (m/m), and digestion was carried out overnight. Dithiothreitol (DTT) was then added to a final concentration of 5 mM, and the samples were reduced at 56 °C for 30 min. Subsequently, iodoacetamide (IAA) was added to a final concentration of 11 mM, followed by incubation for 15 min at room temperature in the dark.

### Enrichment of ubiquitinated peptides

Ubiquitinated peptides were enriched using anti-K-ε-GG antibody–conjugated beads (PTM Biolabs, Hangzhou, China). Peptides were incubated with pre-washed antibody resin in NETN buffer (100 mM NaCl, 1 mM EDTA, 50 mM Tris–HCl, 0.5% NP-40, pH 8.0) at 4 °C overnight with gentle rotation. Beads were washed with NETN and water, and bound peptides were eluted with 0.1% trifluoroacetic acid. Eluted peptides were desalted with C18 ZipTips and dried prior to MS analysis. The enrichment procedure followed the manufacturer’s protocol.

### LC–MS/MS analysis

For the ubiquitinome analysis, peptides were dissolved in mobile phase A and loaded onto Evotips following the manufacturer’s instructions. Peptide separation was performed using an Evosep One UHPLC system with mobile phase A (0.1% formic acid in water) and mobile phase B (0.1% formic acid in acetonitrile). The preset Whisper 40SPD method of the Evosep One system was applied. Separated peptides were introduced into a capillary ion source and analyzed on a timsTOF Pro 2 mass spectrometer (Bruker Daltonics). The ion source voltage was set to 1.5 kV. Both precursor ions and their fragments were detected in TOF mode. Data were acquired in data-independent acquisition parallel accumulation–serial fragmentation (dia-PASEF) mode, with an MS1 scan range of 300–1700 m/z, followed by eight PASEF cycles. MS2 spectra were recorded from 398–1023 m/z using 25 m/z isolation windows.

For the proteome analysis, peptides were dissolved in mobile phase A and separated using a NanoElute UHPLC system (Bruker Daltonics). Mobile phase A consisted of 0.1% formic acid and 2% acetonitrile in water, and mobile phase B consisted of 0.1% formic acid in acetonitrile–water. The chromatographic gradient was set as follows: 6–24% B from 0–14 min, 24–35% B from 14–16 min, 35–80% B from 16–18 min, and 80% B for 18–20 min, at a flow rate of 500 nL/min. Peptides were ionized via a capillary source at 1.75 kV and analyzed on a timsTOF Pro 2 mass spectrometer in TOF mode. Data were acquired in dia-PASEF mode, with an MS1 scan range of 300–1500 m/z, followed by 20 PASEF cycles. MS2 spectra were acquired from 400–850 m/z using 7 m/z windows.

### Mass spectrometry data processing

DIA data were processed using Spectronaut (v18, Biognosys) in directDIA mode. Tandem mass spectra were searched against the *G. lemaneiformis* transcriptome^14^-derived protein database concatenated with a decoy database. This database contained 18,930 predicted protein sequences corresponding to 17,678 genes, with an average of 1.07 transcripts per gene. The completeness of the predicted protein database was evaluated using BUSCO with the eukaryota_odb12 lineage dataset, showing 85.3% complete BUSCOs, including 82.9% single-copy and 2.3% duplicated BUSCOs, with 5.4% fragmented and 9.3% missing BUSCOs. Alternative transcript isoforms were included when multiple transcript models were present for the same gene. Trypsin/P was specified as the proteolytic enzyme, allowing up to two missed cleavages for the proteome dataset and up to four missed cleavages for the ubiquitinome dataset. The higher number of missed cleavages allowed for the ubiquitinome dataset was used to account for the potential impairment of trypsin cleavage efficiency caused by K-ε-GG modification on ubiquitinated lysine residues. Carbamidomethylation of cysteine was set as a fixed modification. For both datasets, N-terminal acetylation and methionine oxidation were defined as variable modifications, whereas lysine ubiquitination (K-ε-GG) was additionally included as a variable modification for the ubiquitinome analysis. The false discovery rate (FDR) at the protein, peptide, and PSM levels was controlled below 1%.

## Data Records

All mass spectrometry data generated in this study, including the proteome and ubiquitinome datasets, have been deposited in the iProX proteomics repository (a partner of the ProteomeXchange Consortium). The data are publicly available under the iProX accession number IPX0014944000^15^ and the corresponding ProteomeXchange accession number PXD072550^16^. Detailed information on dataset, sample name, group, biological replicate, raw file name, and file type is provided in Table 1 to facilitate data retrieval and reuse.

**Table 1:** Sample and raw file information for the proteome and ubiquitinome datasets.

Dataset	Sample name	Group	Biological replicate	Raw file name	File type
Proteome	DAresearch.zip	N/A	N/A	DAresearch.zip	SEARCH
Proteome	DA_CK_1	CK	Replicate 1	XC00928DA_CK_1_Slot2-41_1_18193.d.zip	RAW
Proteome	DA_CK_2	CK	Replicate 2	XC00928DA_CK_2_Slot2-41_1_18194.d.zip	RAW
Proteome	DA_CK_3	CK	Replicate 3	XC00928DA_CK_3_Slot2-41_1_18195.d.zip	RAW
Proteome	DA_LT_1	LT	Replicate 1	XC00928DA_LT_1_Slot2-43_1_18201.d.zip	RAW
Proteome	DA_LT_2	LT	Replicate 2	XC00928DA_LT_2_Slot2-43_1_18202.d.zip	RAW
Proteome	DA_LT_3	LT	Replicate 3	XC00928DA_LT_3_Slot2-43_1_18203.d.zip	RAW
Ubiquitinome	DUPresearch.zip	N/A	N/A	DUPresearch.zip	SEARCH
Ubiquitinome	DPUb_CK_1	CK	Replicate 1	XC00928DPUb_CK_1_S2-B1_1_20047.d.zip	RAW
Ubiquitinome	DPUb_CK_2	CK	Replicate 2	XC00928DPUb_CK_2_S2-B2_1_20048.d.zip	RAW
Ubiquitinome	DPUb_CK_3	CK	Replicate 3	XC00928DPUb_CK_3_S2-B3_1_20049.d.zip	RAW
Ubiquitinome	DPUb_LT_1	LT	Replicate 1	XC00928DPUb_LT_1_S2-B7_1_20055.d.zip	RAW
Ubiquitinome	DPUb_LT_2	LT	Replicate 2	XC00928DPUb_LT_2_S2-B8_1_20056.d.zip	RAW
Ubiquitinome	DPUb_LT_3	LT	Replicate 3	XC00928DPUb_LT_3_S2-B9_1_20057.d.zip	RAW

Note: “DA” denotes the proteome dataset; “DPUb” denotes the ubiquitinome dataset; “CK” denotes the control group; “LT” denotes the low-temperature treatment group; numbers 1–3 indicate biological replicates; “RAW” denotes raw mass spectrometry data files; “SEARCH” denotes database search result files.

**Fig. 2:**
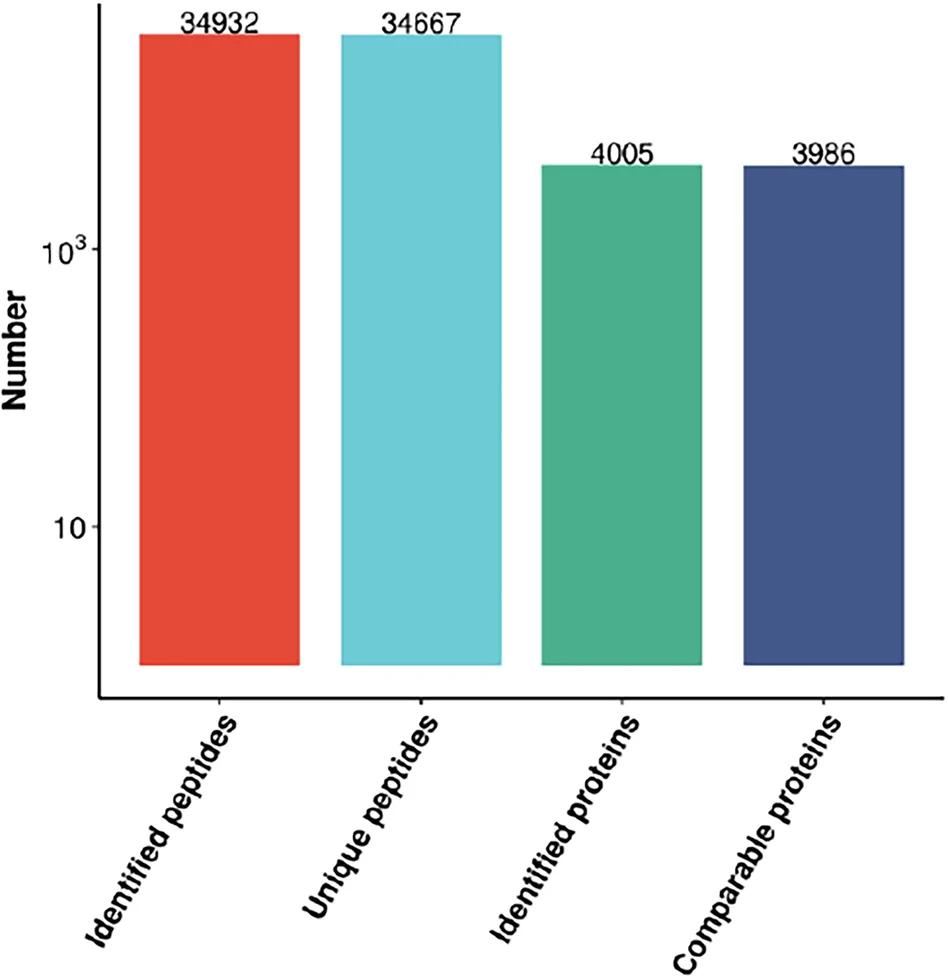
Quantitative summary of the global proteome dataset. The numbers of identified peptides, unique peptides, identified proteins, and comparable proteins are shown.

**Fig. 3:**
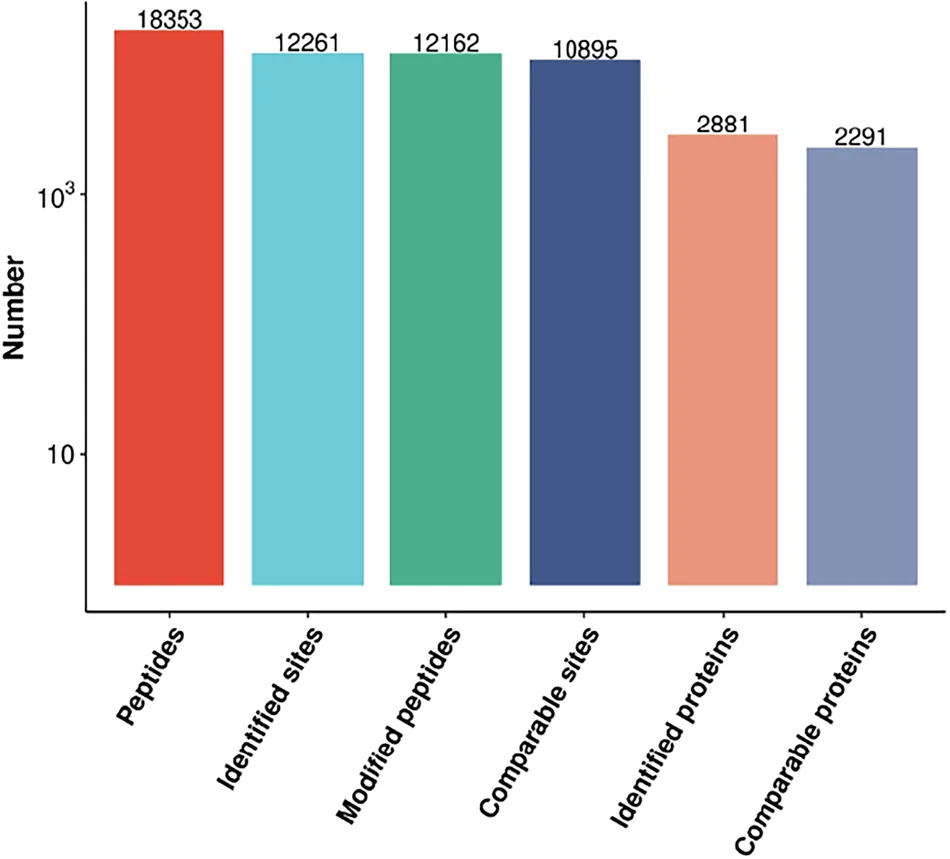
Quantitative summary of the ubiquitinome dataset. The numbers of peptides, identified sites, modified peptides, comparable sites, identified proteins, and comparable proteins are shown.

We added quantitative summary figures (Figs. 2, 3) to present the identification and quantification statistics of the proteome and ubiquitinome datasets, respectively. Tables 2, 3 summarize the numbers of quantified proteins and ubiquitination sites in each biological replicate and the overlap of identified proteins and ubiquitination sites between the CK and LT groups, respectively.

**Table 2:** Numbers of quantified proteins and ubiquitination sites across biological replicates in the proteome and ubiquitinome datasets.

Dataset	CK_1	CK_2	CK_3	LT_1	LT_2	LT_3
Proteome	3,963	3,963	3,966	3,949	3,965	3,951
Ubiquitinome	11,860	11,693	11,584	11,899	11,885	11,866

Note: CK and LT indicate the control and low-temperature treatment groups, respectively; CK_1–CK_3 and LT_1–LT_3 represent biological replicates.

**Table 3:** Overlap of identified proteins and ubiquitination sites between the CK and LT groups.

Dataset	Feature	CK only	Shared between CK and LT	LT only	Total identified proteins/sites
Proteome	Identified proteins	9	3,986	10	4,005
Ubiquitinome	Identified ubiquitination sites	148	11,905	208	12,261

Note: CK and LT indicate the control and low-temperature treatment groups. A protein or ubiquitination site was considered identified in a group if it had a non-zero intensity value in at least one biological replicate. “CK only” and “LT only” indicate features identified exclusively in the CK or LT group, respectively. “Shared between CK and LT” indicates features identified in both groups. “Total identified proteins/sites” represents the union of features identified in CK only, LT only, and shared between both groups.

## Technical Validation

### Quality evaluation of mass spectrometry data

**Fig. 4:**
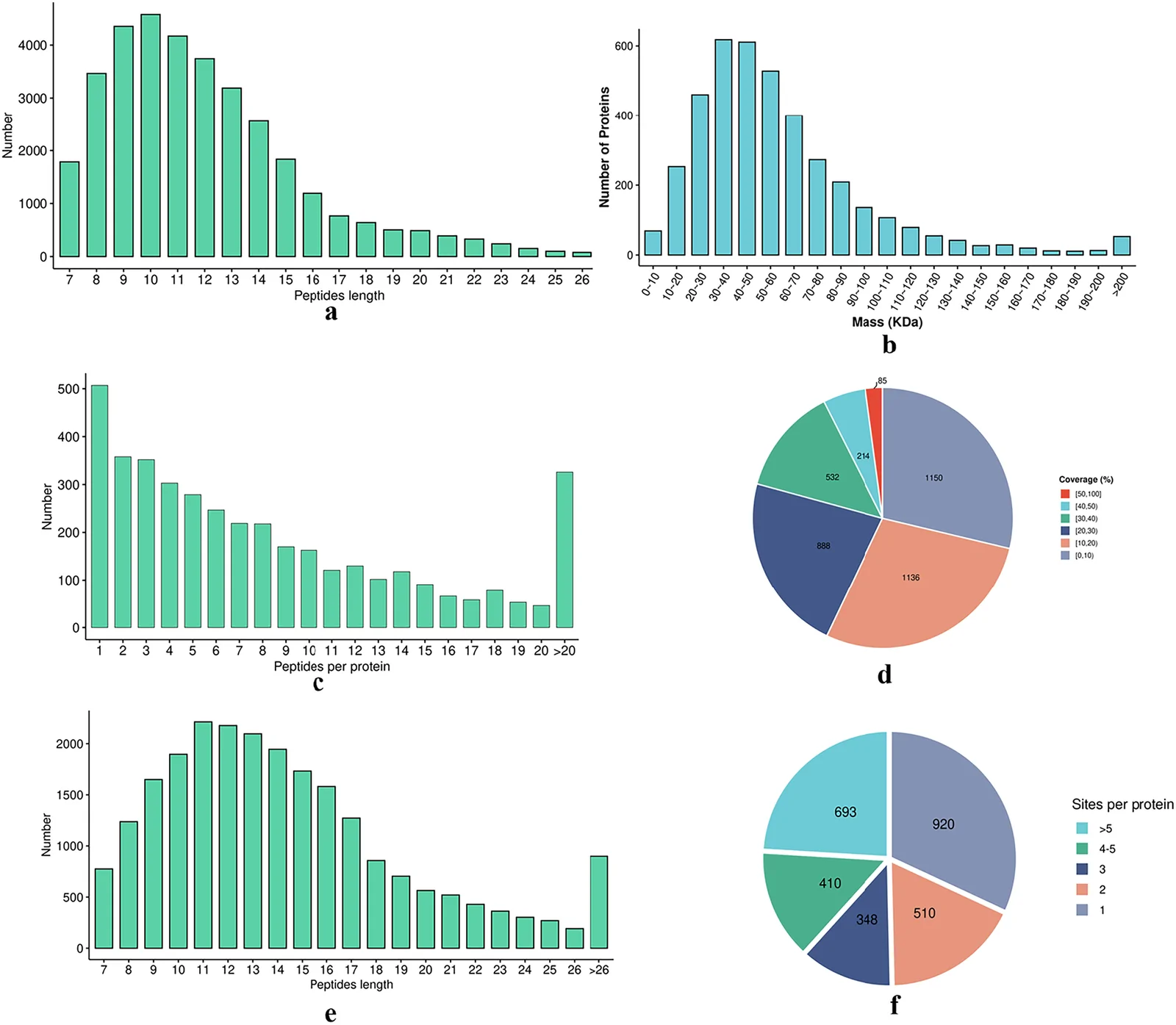
Quality evaluation of the proteome and ubiquitinome mass spectrometry data. (**a**) Peptide length distribution in the proteome dataset. (**b**) Protein molecular weight distribution in the proteome dataset. (**c**) Number of identified peptides per protein in the proteome dataset. (**d**) Protein sequence coverage distribution in the proteome dataset. (**e**) Length distribution of ubiquitinated peptides in the ubiquitinome dataset. (**f**) Distribution of ubiquitination sites per protein in the ubiquitinome dataset. Most identified peptides in the proteome dataset were 8–15 amino acids in length, and most proteins had molecular weights between 20 and 70 kDa. The identified proteins were generally supported by multiple peptides and showed broad sequence coverage. In the ubiquitinome dataset, most ubiquitinated peptides were 9–17 amino acids in length. Among ubiquitinated proteins, 920 proteins contained a single ubiquitination site, whereas a substantial proportion contained multiple sites, including 510 proteins with two sites, 348 proteins with three sites, 410 proteins with four to five sites, and 693 proteins with more than five sites.

To ensure the reliability of our datasets, we performed a quality assessment of both the proteome and ubiquitinome results. As shown in Fig. 4a–d, peptide lengths in the proteome dataset were mainly distributed between 7 and 20 amino acids, protein molecular weights spanned a broad range, and the numbers of identified peptides and sequence coverage per protein showed distributions consistent with typical proteomics datasets, supporting efficient digestion and confident protein identification. For the ubiquitinome (Fig. 4e,f), the peptide-length distribution was comparable, and the distribution of ubiquitination sites per protein was consistent with effective enrichment and site identification. Collectively, these metrics indicate that the mass spectrometry data are of high quality and suitable for downstream analyses.

### Reproducibility of the proteome and ubiquitinome datasets

**Fig. 5:**
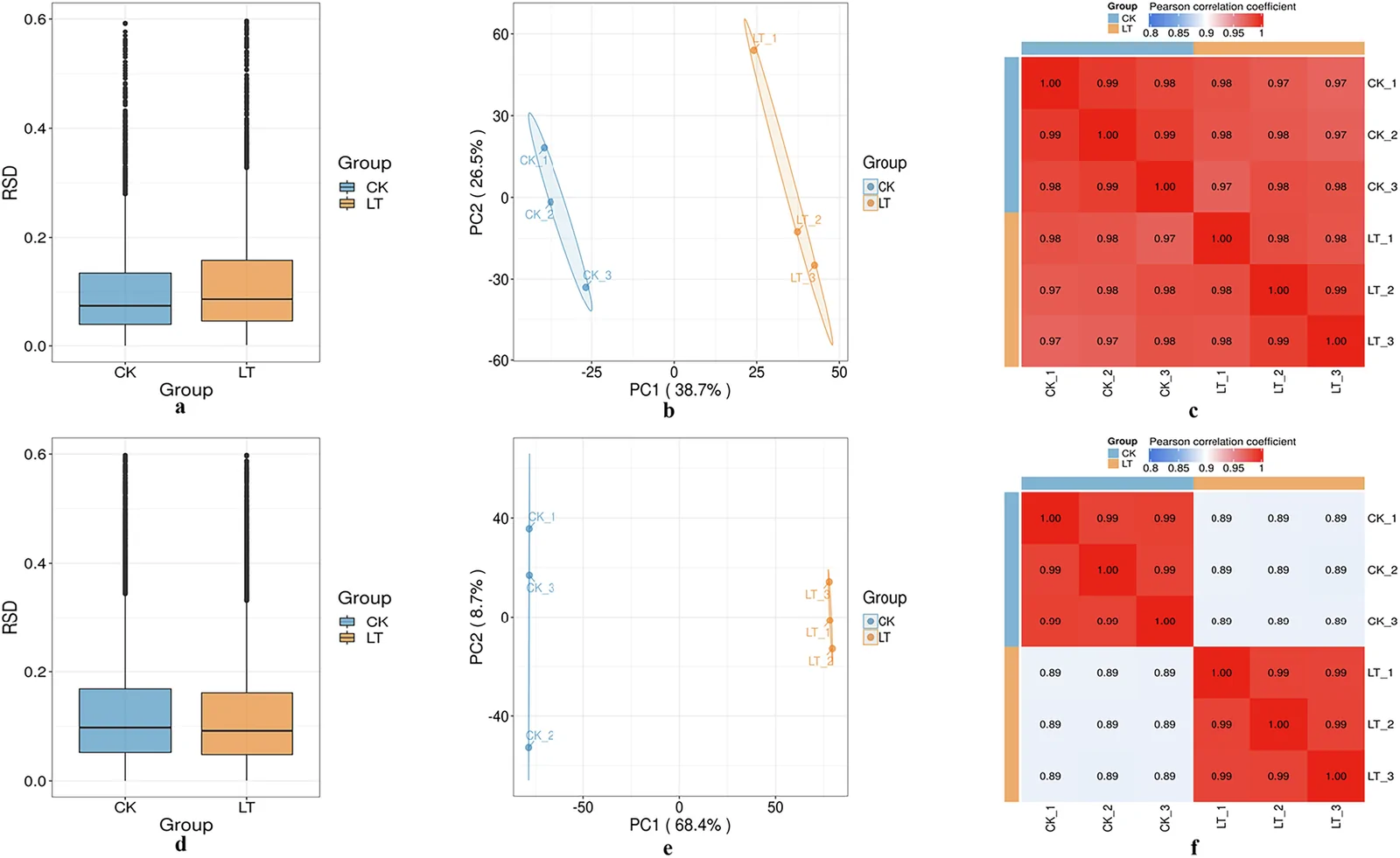
Overview of reproducibility assessments for the proteome and ubiquitinome datasets. Panels (a–c) show the RSD distribution, PCA plot, and Pearson correlation heatmap for the proteome, while panels (d–f) show the corresponding analyses for the ubiquitinome. The RSD distributions indicate overall stable quantitative performance across biological replicates. PCA showed clear separation between CK and LT samples in both datasets, indicating distinct proteomic and ubiquitinomic responses to low-temperature treatment. Pearson correlation analysis showed high correlations among biological replicates, supporting good reproducibility of both datasets.

We assessed the reproducibility of the proteome and ubiquitinome data by calculating the relative standard deviation (RSD) across three biological replicates. All samples exhibited RSD values below 0.2, indicating a high degree of consistency among replicates (Fig. 5a,d). The proteome showed lower RSD values than the ubiquitinome, which is consistent with the higher variability often observed for post-translational modification datasets. PCA analysis further supported reproducibility (Fig. 5b,e): biological replicates within each group clustered closely together, and the CK and LT groups were separated in the PCA space, indicating a detectable global response to low-temperature treatment. Pearson correlation analysis showed that within-group correlations were consistently higher than between-group correlations for both datasets (Fig. 5c,f), supporting the overall robustness of quantitative measurements. Collectively, these quality statistics indicate that both datasets have high reproducibility and are suitable for downstream analyses.

## Data Availability

The mass spectrometry proteomics data have been deposited in the ProteomeXchange Consortium through the iProX partner repository^17,18^, with the ProteomeXchange accession number PXD072550 and the iProX accession number IPX0014944000.

## Code availability

Data analysis procedures have been described in detail in the Methods section. No custom code was used during this study for the curation and/or validation of the dataset.
